# CXCL11 Correlates with Immune Infiltration and Impacts Patient Immunotherapy Efficacy: A Pan-Cancer Analysis 

**DOI:** 10.3389/fimmu.2022.951247

**Published:** 2022-07-22

**Authors:** Yang Li, Shukun Han, Baokang Wu, Chongli Zhong, Yu Shi, Chao Lv, Lei Fu, Yizhou Zhang, Qi Lang, Zhiyun Liang, Yang Yu, Yu Tian

**Affiliations:** ^1^ Department of General Surgery, Shengjing Hospital of China Medical University, Shenyang, China; ^2^ Department of General Surgery, The First Affiliated Hospital of China Medical University, Shenyang, China; ^3^ Department of General Surgery, The First Affiliated Hospital of Jinzhou Medical University, Jinzhou, China

**Keywords:** chemokine ligand C-X-C motif chemokine ligand 11, pan-cancer analysis, immunotherapy, tumor immunity, tumor microenvironment

## Abstract

**Background:**

Immunotherapy has achieved great success in cancer. Nevertheless, many patients cannot benefit from immune checkpoint blockade therapy because of the scantiness of CD8+ T cell infiltration in the tumor microenvironment (TME). CXCL11 is known as a regulator that influences T-cell infiltration into tumors. However, the role of CXCL11 in pan-cancer is still unclear.

**Methods:**

In this study, we investigated the expression and function of CXCL11 across 33 types of cancers based on datasets from The Cancer Genome Atlas (TCGA) database and the Genotype-Tissue Expression (GTEx) database. We analyzed the CXCL11 differential expression in tumor tissue and nontumoral tissue and in different stages of cancers. Moreover, the correlations among CXCL11 expression, prognosis, mismatch repair, tumor mutation burden (TMB), microsatellite instability (MSI), tumor microenvironment, and immune-related genes were evaluated.

**Results:**

CXCL11 expression was significantly higher in tumoral tissue than in nontumoral tissue for most types of cancer. Improved CXCL11 expression was related to an inconsistent prognosis in different cancers. CXCL11 was positively associated with CD8+ T cells and T follicular helper cells in the TME. High expression of CXCL11 was positively related to TMB in BLCA, BRCA, CESC, COAD, LGG, LUAD, OV, SKCM, STAD, THYM, and UCEC. A positive correlation between CXCL11 and MSI was found in COAD and UVM. Moreover, functional analysis of CXCL11 showed that high CXCL11 expression was significantly related to immune-relevant pathways.

**Conclusions:**

CXCL11 might function as a prognostic and immunotherapy marker across cancers. Further investigation into CXCL11 might provide promising insights to improve cancer therapy.

## Introduction

Cancer immunotherapy has become a promising cancer treatment option in recent years. (1) In addition, immune checkpoint inhibitors (ICIs) significantly benefit patients with many solid tumors. Despite this circumstance, a large portion of patients still cannot obtain a satisfactory therapeutic effect from immune checkpoint blockade therapy (2, 3).

To date, mounting evidence has demonstrated that the efficacy of ICIs is dependent on a sturdy antitumor immune response, and improving CD8+ T-cell infiltration into the tumor is key to promote antitumor immunity (4, 5). Moreover, it has been widely accepted that the absence of effector T-cell infiltration in the TME is one of the main reasons that ICI therapies do not respond well (6). It is indispensable to improve the ICI therapy response by increasing CD8+ T-cell infiltration into the TME.

Chemokines are small proteins that can act as a ‘ZIP code’ for T cells and other immune cells (7), which can mediate the trafficking of immune cells into the TME by binding to the 7-transmembrane G-protein-coupled receptor (GPCR) family. (8, 9) Among the numerous cytokines, a type of cytokine called CXCR3 ligands contains CXCL9, CXCL10, and CXCL11 (10), which can effectively recruit CD8+ T-cell infiltration into the TME (11). Therefore, CXCL9, CXCL10, and the CXCL11/CXCR3 axis have been regarded as potential cancer immune therapy targets (12). Among the three CXCR3 ligands, CXCL11 shows the highest affinity for CXCR3 compared to CXCL9 and CXCL10. (13) Despite this circumstance, in contrast to CXCL9 and CXCL10, several reports have shown that CXCL11 increases cancer cell aggressiveness, migration, and tumor metabolism in certain cancers (14, 15). Hence, the role of CXCL11 in tumors deserves to be further investigated.

CXCL11, also known as interferon-inducible T-cell alpha chemoattractant (I-TAC), in addition to binding to classical GPCRs, has been reported to be the ligand of specific atypical chemokine receptors (ACKRs), including CXCR7(ACKR3) (10), and GPR182 (16), to date. Remarkably, the ACKR family, which is part of the superfamily of GPCRs, has been demonstrated to be expressed on stromal cell types and participate in cell migration regulation in developmental, inflammatory, and pathological conditions. ACKR family functions through scavenging, transporting, or storing chemokines. as well as by regulating shared ligand canonical chemokine receptor activity to influence the chemokine system (17). CXCR7 has been reported to be upregulated in expression and is related to the progression of specific tumors (18), while CXCR3 has been shown to have at least three variants, CXCR3A, CXCR3B, and CXCR3-alt, with each exerting unique functions (12, 19). Because of the diversity of receptors and the variants of its CXCR3 receptor, CXCL11 might contribute to tumor progression in certain types of cancers. Therefore, the role of CXCL11 across cancers is still unclear.

The association of CXCL11 with cancers has not been studied, and several research studies about the role of CXCL11 in tumors have been limited to only specific types of cancer such as colon cancer. Therefore, we analyzed data from TCGA and the GTEx databases to investigate CXCL11 expression levels and their relationship with prognosis across cancers. We also analyzed the association between CXCL11 expression and TMB, MSI, TME, immune-related genes, and immune infiltration to shed light on the role of CXCL11 in 33 types of tumors (**Table 1**). The present study discovered that CXCL11 expression was upregulated in most tumors. Additionally, the level of CXCL11 was closely correlated with prognosis, TMB, MSI, immune checkpoints, TME, immune cell infiltration, and immune-related genes, providing insights to the role of CXCL11 in various cancer types.

**Table 1:** TCGA cancer abbreviations and the corresponding cancer type.

Abbreviations	Cancer Type
ACC	Adrenocortical carcinoma
BLCA	Bladder Urothelial Carcinoma
BRCA	Breast invasive carcinoma
CESC	Cervical squamous cell carcinoma and endocervical adenocarcinoma
CHOL	Cholangiocarcinoma
COAD	Colon adenocarcinoma
DLBC	Lymphoid Neoplasm Diffuse Large B-cell Lymphoma
ESCA	Esophageal carcinoma
GBM	Glioblastoma multiforme
HNSC	Head and Neck squamous cell carcinoma
KICH	Kidney Chromophobe
KIRC	Kidney renal clear cell carcinoma
KIRP	Kidney renal papillary cell carcinoma
LAML	Acute Myeloid Leukemia
LGG	Brain Lower Grade Glioma
LIHC	Liver hepatocellular carcinoma
LUAD	Lung adenocarcinoma
LUSC	Lung squamous cell carcinoma
MESO	Mesothelioma
OV	Ovarian serous cystadenocarcinoma
PAAD	Pancreatic adenocarcinoma
PCPG	Pheochromocytoma and Paraganglioma
PRAD	Prostate adenocarcinoma
READ	Rectum adenocarcinoma
SARC	Sarcoma
SKCM	Skin Cutaneous Melanoma
STAD	Stomach adenocarcinoma
TGCT	Testicular Germ Cell Tumors
THCA	Thyroid carcinoma
THYM	Thymoma
UCEC	Uterine Corpus Endometrial Carcinoma
UCS	Uterine Carcinosarcoma
UVM	Uveal Melanoma

## Materials and Methods

### Data Collection

The RNA expression and somatic mutation data were downloaded from the TCGA (https://portal.gdc.cancer.gov/) by UCSC Xena (http://xena.ucsc.edu/). Additionally, we used data from the GTEx database (https://www.gtexportal.org/home/).

### Expression Analysis

Data downloaded from TCGA and GTEx databases were utilized to evaluate the gene expression fo CXCL11 across 31 normal tissues and 33 tumor tissues. All expression data were log2 transformed. Differential expression with P < 0.05 was considered significant between tumor and normal tissues.

### Survival Analysis and ROC Analysis

Kaplan–Meier analysis was used to analyze the survival difference between the high expression and low expression groups to evaluate the prognostic value of CXCL11. Survival curves were drawn by the survival and survminer R packages. The prognostic values between CXCL11 expression and overall survival (OS), disease-specific survival (DSS), disease-free interval (DFI), and disease-free interval (PFI) across the cancers studied was evaluated by univariate Cox analysis. The survival results included the hazard ratio (HR), 95% confidence intervals (CI), and p value. We also used the rms R package to plot time-dependent receiver operating characteristic (ROC) curves across the cancers studied.

### Analysis of MMR genes, TMB, and MSI in Cancers

Expression profile data from TCGA were used to evaluate the correlation between the expression levels of CXCL11 and MMR genes, which contained the MutL homologous gene (MLH1), MutS homologous gene (MSH2, MSH6), increased separation after meiosis (PMS2), and epithelial cell adhesion molecule (EPCAM). TMB is a quantitative measure of the total number of somatic nonsynonymous mutations per coding area of a tumor genome (20) and is regarded as a promising immune-response biomarker (21). MSI, a result of mismatch repair deficiency (22), is another biomarker to predict the PD-1 therapy response in solid tumors (23). The Spearman’s rank correlation coefficient was used to analyze the association between CXCL11 expression and TMB as well as MSI .

### TME and Immune Infiltration

The TME consists of various cell types such as immune cells and extracellular components, such as cytokines around the tumor cells, which could affect therapy effectiveness and clinical outcomes (24). The CXCL11 expression profile and the abundance of tumor-infiltrating immune cells (TIICs) across cancers were analyzed using the the TIMER 2.0 database (http://timer.cistrome.org/) (25). We also analyzed the correlation between CXCL11 and immune-related genes, including MHCs, immunostimulatory genes, immunosuppressive genes, chemokine receptor proteins, and other chemokines, across cancers.

### Enrichment Analysis

Gene set enrichment analysis (GSEA) was performed to examine the pathways affected by CXCL11 in tumors. The GSVA gene set was downloaded from the MSigDB database (https://www.gsea-msigdb.org/gsea/msigdb/index.jsp; v7.5.1 updated January 2022). The entire biological process was evaluated according to the Kyoto Encyclopedia of Genes and Genomes (KEGG) and HALLMARK pathways.

### Statistical Analysis

All the gene expression data were normalized by log2 [TPM (Transcripts per million) +1] transformation. Student’s t-test was used for comparisons between two groups. For comparisons among >2 groups, Kruskal–Wallis test was used as a non-parametric method and adopted the one-way ANOVA test as a parametric method. The correlation analysis between the two variables used Spearman’s test; P < 0.05 was considered significant.The R software(Version 4.1.3) was used in this analysis.

## Results

### CXCL11 Expression Analysis in Pan-Cancer

**Figure 1:**
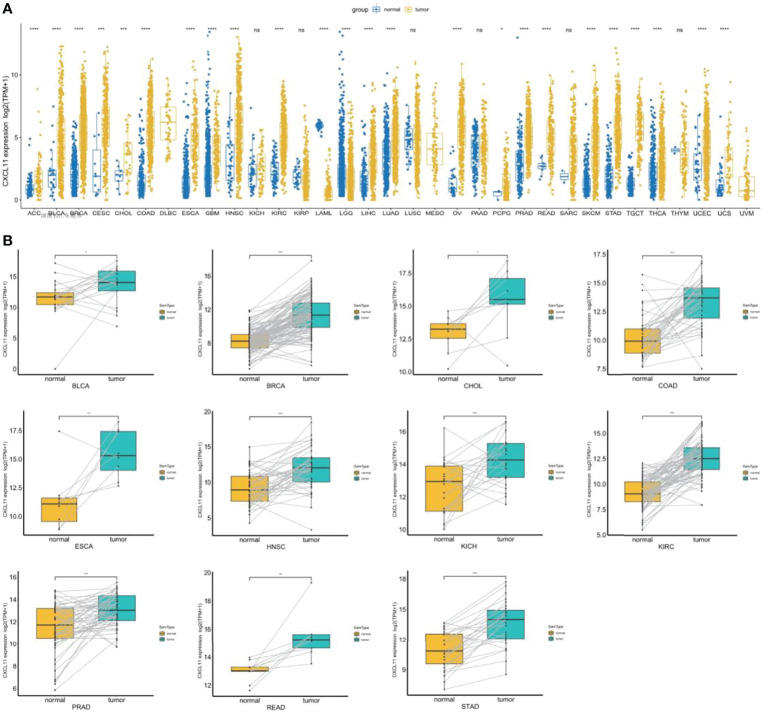
CXCL11 profile across cancers. **(A)** The expression of CXCL11 across normal and tumor tissues in the integrated GTEx database and TCGA database. **(B)** According to TCGA data, the levels of CXCL11 in corresponding tumors and normal tissues in BLCA, BRCA, CHOL, COAD, ESCA, HNSC, KICH, KIRC, PRAD, READ, and STAD. ns denotes not statistically significant, *p < 0.05, **p < 0.01, ***p <0.001, ****p < 0.0001.

To explore the expression of CXCL11, we analyzed the mRNA expression across cancers by integrating the GTEx and TCGA databases. CXCL11 was differentially expressed in ACC, BLCA, BRCA, CESC, CHOL, COAD, ESCA, GBM, HNSC, KIRC, LAML, LGG, LIHC, LUAD, OV, PCPG, PRAD, READ, SKCM, STAD, TGCT, THCA, UCEC, and UCS in the TCGA cohort. Increased expression was found in ACC, BLCA, BRCA, CESC, CHOL, COAD, ESCA, GBM, HNSC, KIRC, LIHC, LUAD, OV, PCPG, PRAD, READ, SKCM, STAD, TGCT, THCA, UCEC, and UCS, while decreased expression was found in LAML, and LGG (**Figure 1A**). Next, we used paired expression analysis to evaluate the expression of CXCL11 in corresponding tumors and nontumoral tissues. The data showed that the expression of CXCL11 was significantly upregulated in 12 types of tumors, including BLCA, BRCA, CHOL, COAD, ESCA, HNSC, KICH, KIRC, PRAD, READ, and STAD (**Figure 1B**).

### Analysis of the Relationship Between CXCL11 Expression and Tumor Stages

**Figure 2:**
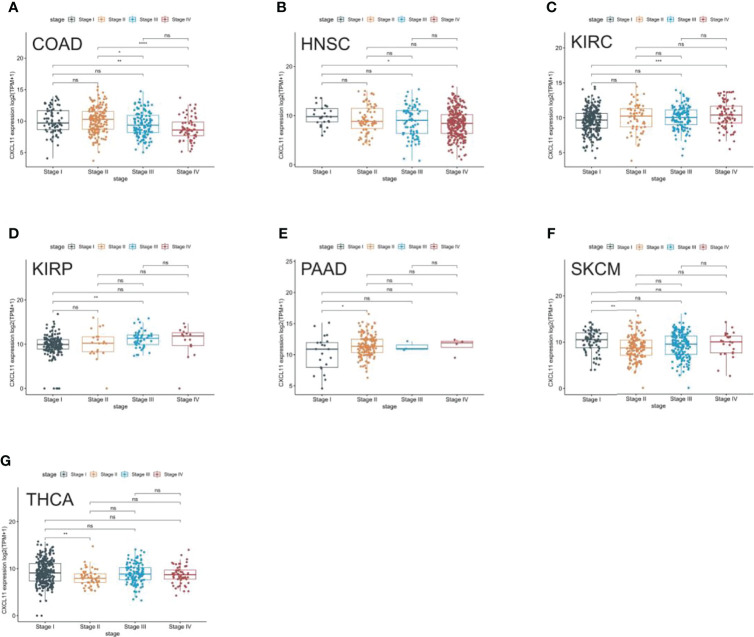
Pan-cancer CXCL11 expression in different cancer stages. **(A–G)** Comparison of CXCL11 expression in different cancer stages defined by the World Health Organization of different tumors in COAD, HNSC, KIRC, KIRP, PAAD, SKCM, and THCA. ns denotes not statistically significant, *p < 0.05, **p < 0.01, ***p <0.001, ****p < 0.0001.

We analyzed the expression of CXCL11 in different stages of 33 tumors and found that CXCL11 expression was closely associated with tumor stage in 7 types of cancer, including COAD, HNSC, KIRC, KIRP, PAAD, SKCM, and THCA (**Figure 2**). Remarkably, the closest association between the tumor stage and the expression of CXCL11 occurred in stage I and other stages. Interestingly, CXCL11 expression increased from stage I to other stages in KIRC, KIRP, and PAAD, while it decreased from stage I to other stages in CIAD, HNSC, SKCM and THCA. Differential expression was also found in stage II compared to stage III and IV in COAD. However, the expression of CXCL11 was not significantly different between different tumor stages in the remaining cancers.

### Prognostic Value of CXCL11 Across Cancers

**Figure 3:**
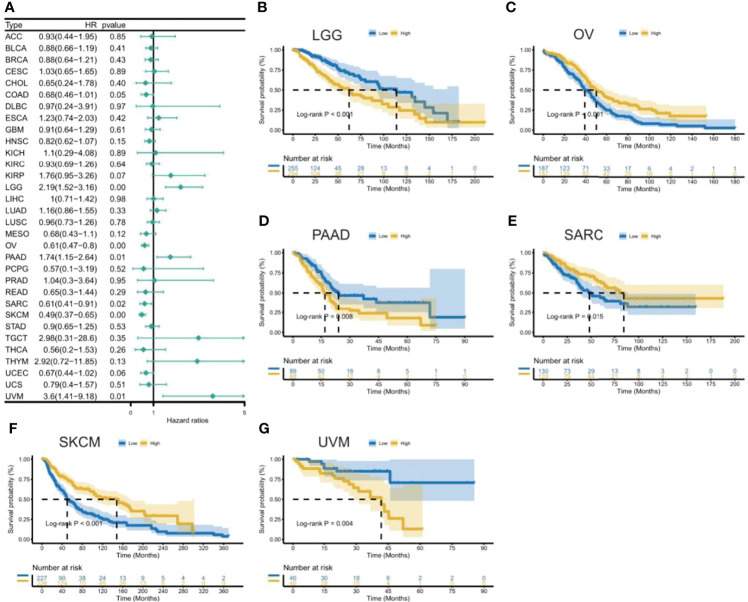
Association between CXCL11 expression and overall survival (OS). **(A)** Forest plot of OS association across cancers. **(B–G)** Kaplan–Meier analysis of the association between CXCL11 expression and OS in LGG, OV, PAAD, SARC, SKCM, and UVM.

**Figure 4:**
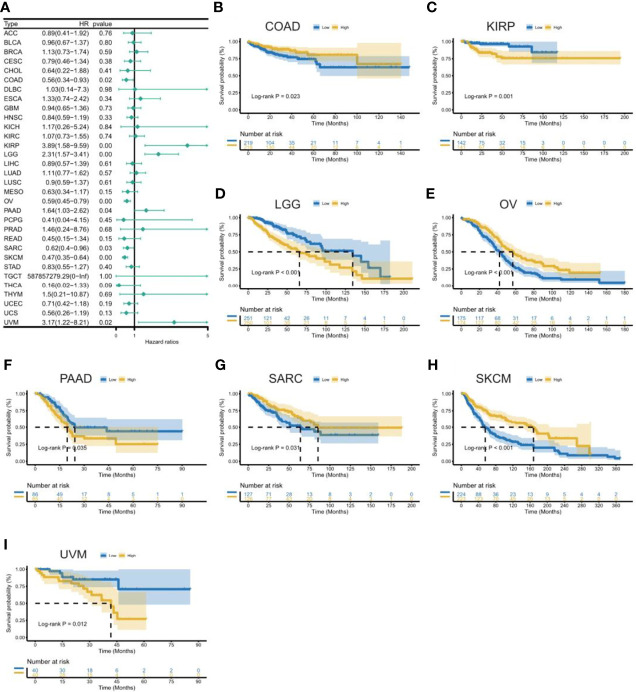
Association between CXCL11 expression and disease-specific survival (DSS). **(A)** Forest plot of DSS association with CXCL11 expression across cancers. **(B–I)** Kaplan–Meier analysis of the association between CXCL11 expression and DSS in COAD, KIRP, LGG, OV, PAAD, SARC, SKCM, and UVM.

**Figure 5:**
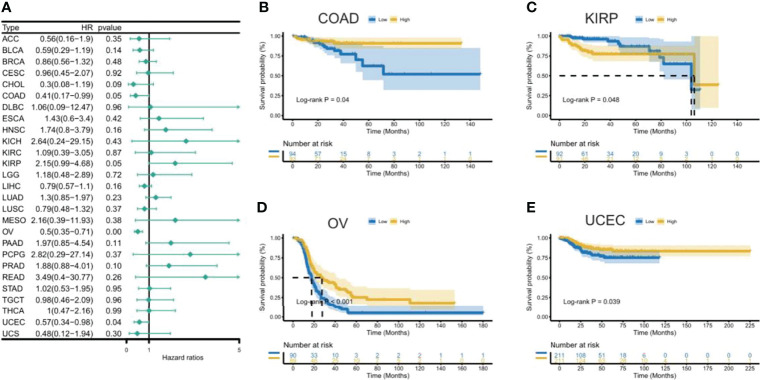
Association between CXCL11 expression and disease-free interval (DFI). **(A)** Forest plot of DFI association with CXCL11 expression across cancers. **(B–E)** Kaplan–Meier analysis of the association between CXCL11 expression and DFI in COAD, KIRP, OV, and UCEC.

**Figure 6:**
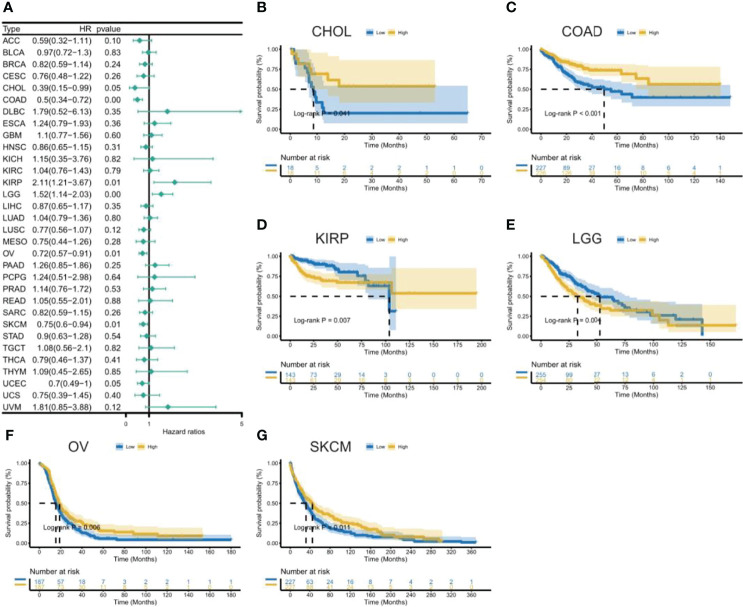
Association between CXCL11 expression and disease-free interval (PFI). **(A)** Forest plot of DFI association with CXCL11 expression across cancers. **(B–G)** Kaplan–Meier analysis of the association between CXCL11 expression and DFI in CHOL, COAD, KIRP, LGG, OV, and SKCM.

**Figure 7:**
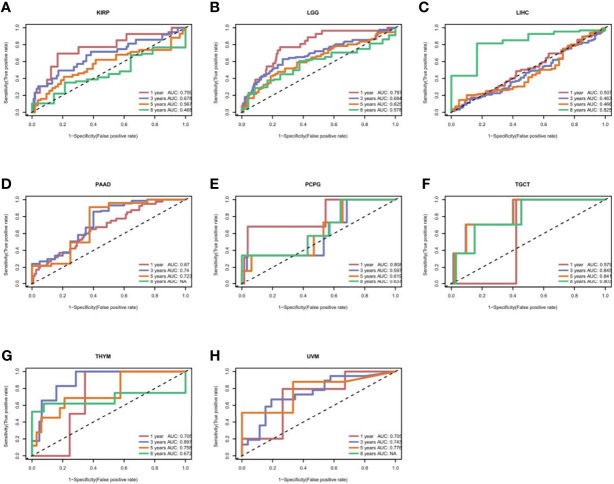
The time-dependent receiver operating characteristic (ROC) curves for KIRP **(A)**, LGG **(B)**, LIHC **(C)**, PAAD **(D)**, PCPG **(E)**, TGCT **(F)**, THYM **(G)**, and UVM **(H)**. Area under the curve (AUC) scores in evaluating 1-, 3-, 5-, and 8-year OS are also shown in the figures.

To uncover the association between the expression of CXCL11 and prognosis, we performed survival association analysis, including overall survival (OS), disease-specific survival (DSS), disease-free interval (DFI), and progression-free interval (PFI), across 33 types of cancers in TCGA. Cox proportional hazards model analysis showed that CXCL11 expression was associated with COAD, LGG, OV, PAAD, SARC, SKCM, and UVM (**Figure 3A**). The Kaplan–Meier (KM) curves for tumors in which CXCL11 expression was significantly associated with the patient outcome are shown in **Figures 3** **B–G**. The results showed that high expression of CXCL11 was associated with poor prognosis in patients with LGG, PAAD, and UVM; however, in patients with OV, SARC, and SKCM high CXCL11 expression was associated with better prognosis. Moreover, Cox proportional hazards model analysis of DSS data (**Figure 4A**) showed that CXCL11 expression was associated with COAD, KIRP, LGG, OV, PAAD, SARC, SKCM, and UVM. Kaplan–Meier survival analysis revealed a correlation between high CXCL11 expression levels and poor prognosis in patients with KIRP, LGG, PAAD, and UVM. However, the opposite relationship was observed in patients with COAD, OV, SARC, and SKCM. Cox proportional hazards model analysis of DFI data (**Figure 5A**) showed that CXCL11 expression was associated with COAD, KIRP, OV, and UCEC. Kaplan–Meier survival analysis revealed a correlation between high CXCL11 expression levels and poor prognosis in patients with KIRP. However, the opposite relationship was observed in patients with COAD, OV, and UCEC. Cox proportional hazards model analysis of PFI data (**Figure 6A**) showed that CXCL11 expression was associated with CHOL, COAD, KIRP, LGG, OV, and SKCM. Kaplan–Meier survival analysis revealed a correlation between high CXCL11 expression levels and poor prognosis in patients with KIRP and LGG. However, the opposite relationship was observed in patients with CHOL, COAD, OV, and SKCM. Time-dependent ROC curves were also plotted across different cancers. The results, including KIRP, LGG, LIHC, PAAD, PCPG, TGCT, THYM, and UVM, are shown in **Figure 7**. Time-dependent ROC curves showed that the CXCL11 expression model had an area under the curve (AUC) value of 0.759 in evaluating 1 year OS in KIRP, an AUC value of 0.787 in evaluating 1 year OS in LGG, an AUC value of 0.825 in evaluating 8 years OS in LIHC, an AUC value of 0.74 and 0.723 in evaluating 3 years and 5 years OS in PAAD, respectively, an AUC value of 0.808 in evaluating 1 year OS in PCPG, an AUC value of 0.845, 0.841, and 0.803 in evaluating 3 years, 5 years, and 8 years OS in TGCT, respectively, an AUC value of 0.705, 0.897, and 0.758 in evaluating 1 year, 3 years, and 5 years OS in THYM, respectively, and an AUC value of 0.705, 0.743, and 0.776 in evaluating 1 year, 3 years, and 5 years OS in UVM, respectively (**Figure 7**).

### Relationship of CXCL11 Expression With MMR genes, TMB, and MSI

In MMR-deficient cancers, the large proportion of mutant neoantigens makes them sensitive to immune checkpoint blockade, regardless of their tissue of origin (26). TMB (27) and MSI (28) are also recognized biomarkers that reflect the treatment effect of ICB therapy.

**Figure 8:**
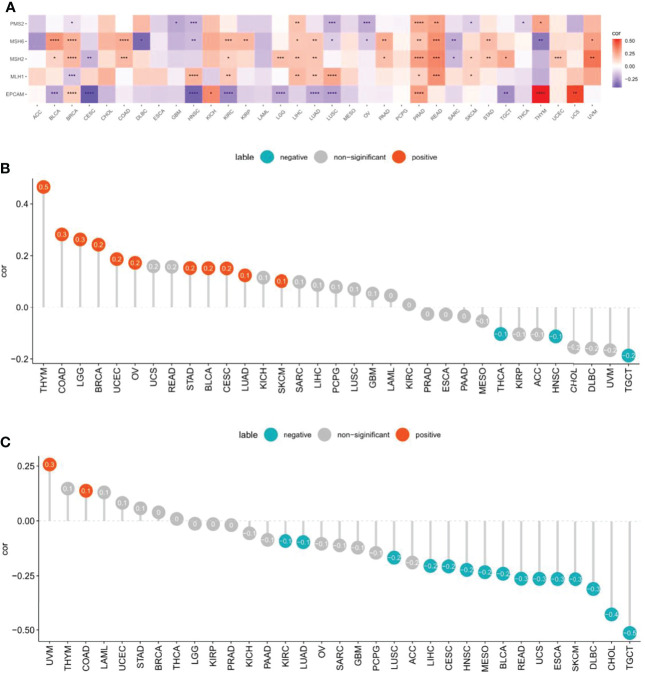
Associations between CXCL11 expression and mismatch repair (MMR), tumor mutational burden (TMB), and microsatellite instability (MSI) across cancers. **(A)** Association between CXCL11 expression and MMR genes in pan-cancer by heatmap. *p < 0.05, **p < 0.01, ***p < 0.001, and ****p < 0.0001 **(B)** Association between CXCL11 expression and TMB across cancers. **(C)** Association between CXCL11 expression and MSI across cancers.

We assessed whether there were correlations between CXCL11 expression and MMR genes, including MLH1, MSH2, MSH6, PMS2, and EPCAM. We analyzed the relationship between CXCL11 and MMR genes and displayed the results *via* a heatmap. The results showed that CXCL11 expression was closely related to MMR genes in 12 cancers, including BLCA, BRCA, HNSC, KIRC, LIHC, LUAD, LUSC PRAD, READ, SKCM, THYM, and UVM (**Figures 8A**). Then, we evaluated the association between TMB and CXCL11 expression. The results showed that CXCL11 expression was positively related to TMB in BLCA, BRCA, CESC, COAD, LGG, LUAD, OV, SKCM, STAD, THYM, and UCEC but negatively related to TMB in HNSC, TGCT, and THCA (**Figure 8B**). The correlation between CXCL11 and MSI was also checked in our study. The analysis showed that CXCL11 expression was positively related to MSI in COAD and UVM but negatively related to MSI in BLCA, CESC, CHOL, DLBC, ESCA, HNSC, KIRC, LIHC, LUAD, LUSC, MESO, READ, SKCM, TGCT, and UCS (**Figures 8C**).

### Correlation Between CXCL11 Expression and Tumor Purity

**Figure 9:**
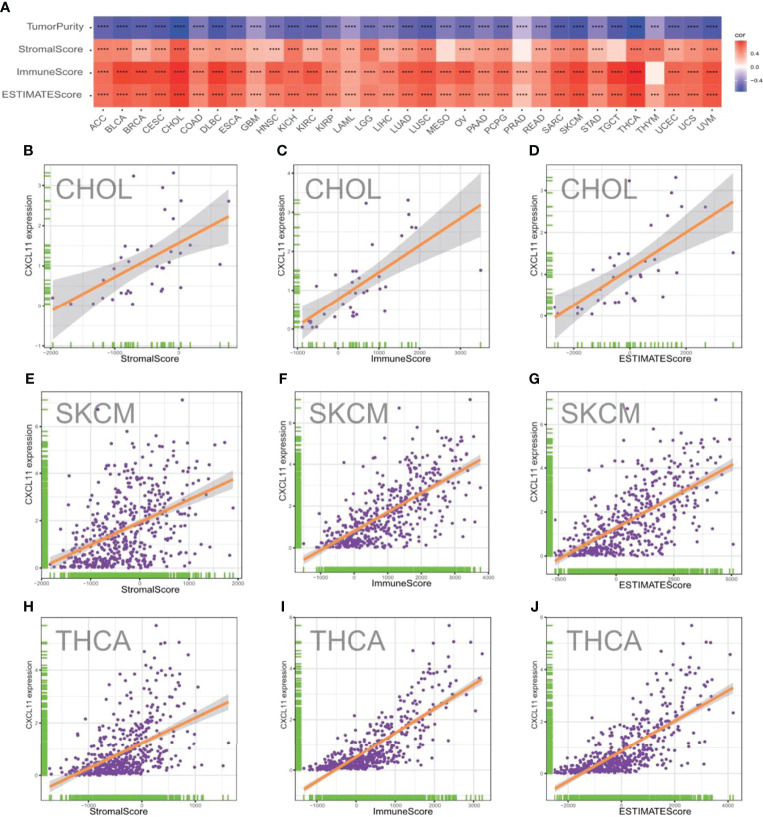
Correlation between CXCL11 expression and ImmuneScore and StromalScore in pam-cancer. **(A)** Spearman’s correlation analysis of CD93 expression with ImmuneScore, StromalScore, and EstimateScore in pan-cancer (*p < 0.05, **p < 0.01, ***p < 0.001, ****p < 0.0001). Correlation between CXCL11 expression and three scores in CHOL **(B–D)**, SKCM **(E–G)**, and THCA **(H–J)**.

The TME, which contains stromal cells, fibroblasts, endothelial cells, innate immune cells, and adaptive immune cells, is a complex system. Understanding the TME might help improve the outcome of therapy (29). To evaluate the correlation between CXCL11 expression and features of the TME, we calculated the tumor purity, stromal scores, immune scores, and ESTIMATE scores across different cancers (**Figure 9A**). As shown in **Figure 9A**, the expression of CXCL11 was positively related to the StromalScore, ImmuneScore, and EstimateScore in the vast majority of cancers with statistical significance. However, the results showed that CXCL11 expression was negatively related to tumor purity in all 33 cancers. The results of CHOL, SKCM, and THCA in detail are visualized and shown in **Figures 9** **B–J**.

### Correlation Between CXCL11 Expression and Immune Infiltration and Immunoregulation Related Genes in Pan-Cancer Analysis

**Figure 10:**
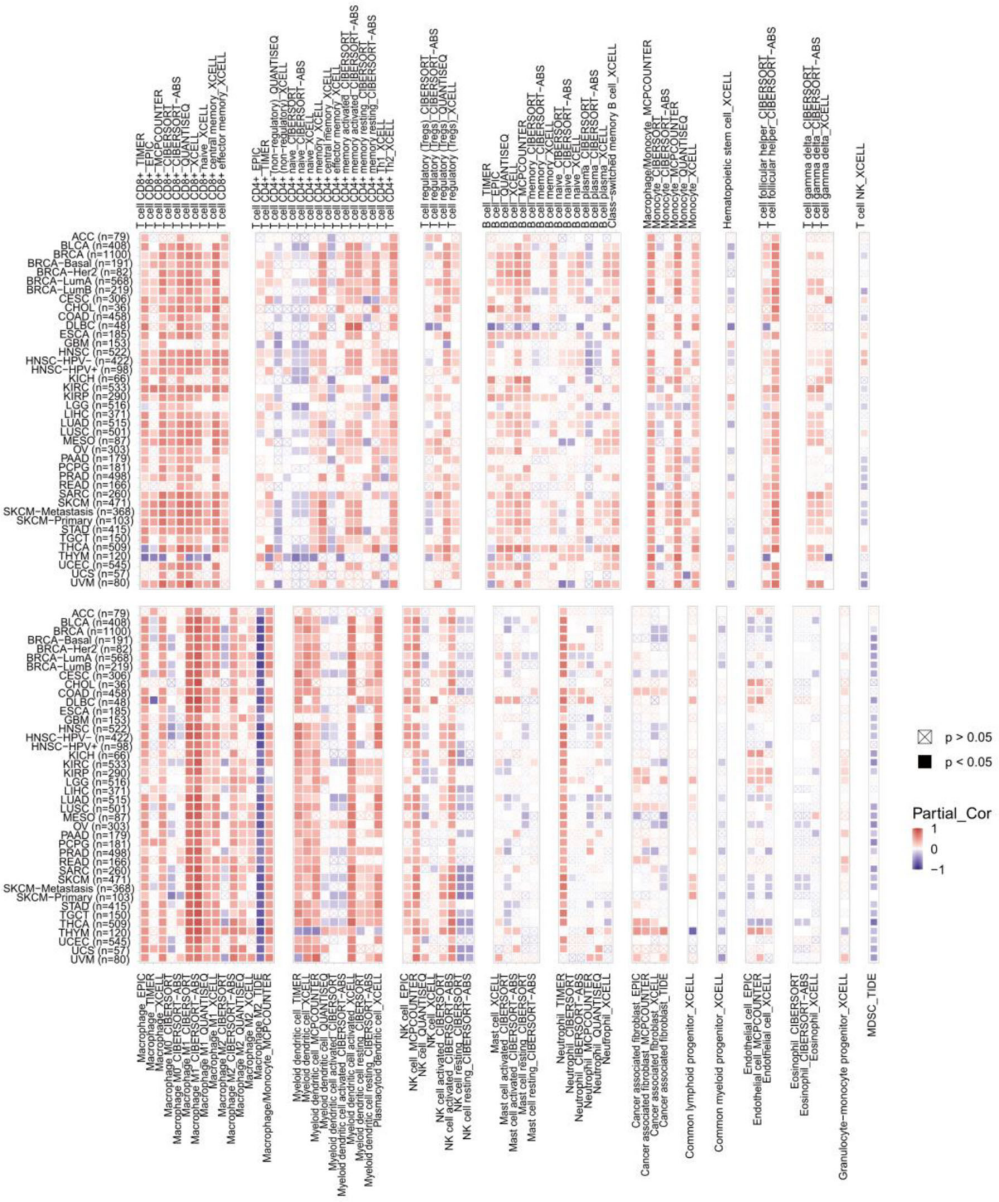
The correlation between CXCL11 expression and immune infiltration across cancers.

**Figure 11:**
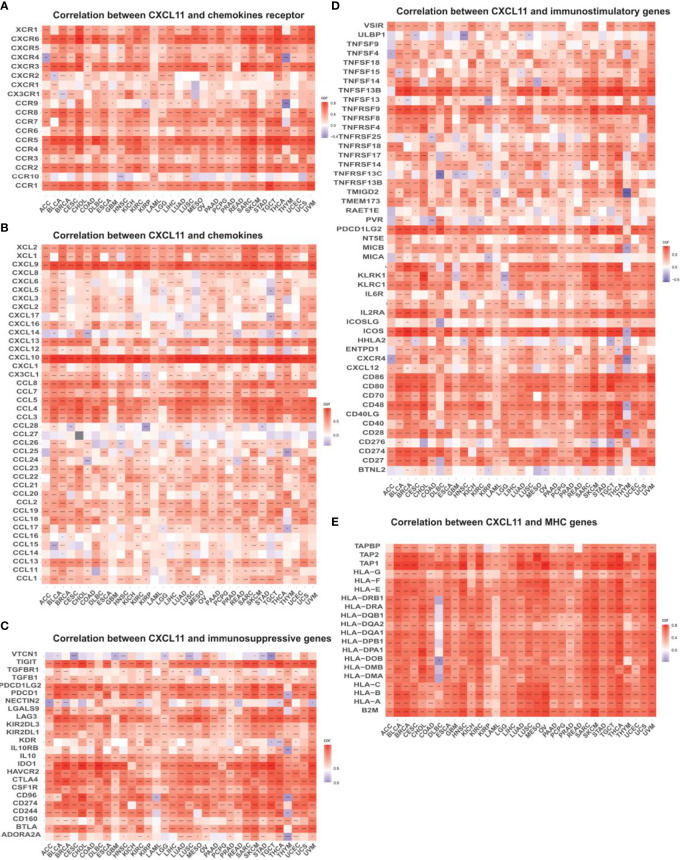
Relationship between CXCL11 and **(A)** chemokine receptors, **(B)** chemokines, **(C)** immunosuppressive factors, **(D)** immunostimulatory factors, and **(E)** MHC genes. *p < 0.05, **p < 0.01, ***p < 0.001, ****p < 0.0001.

To explore the relevance between CXCL11 expression and immune infiltration across cancers, we used the TIMER 2.0 database to investigate the correlation between CXCL11 expression and infiltrating immune cells. Our results revealed that CXCL11 expression was positively associated with CD8+ T cells and T follicular helper cells but negatively related to MDSCs in almost all cancers (**Figure 10**). Then we checked the relationship between CXCL11 expression and immune-related genes, including chemokine receptors, other chemokines, immunostimulatory genes, immunosuppressive genes, and MHC genes, across all 33 cancers. As shown in **Figure 11**, CXCL11 was positively related to approximately all immune-related genes in almost all cancers. We analyzed the association between CXCL11 and chemokine receptors as well as other chemokines (**Figures 11** **A, B**). The results revealed that CXCL11 was positively related to almost all chemokine receptors in almost all cancers except LAML. Moreover, the correlation was also found in all chemokine receptors except CXCR1 and CCR10. For other chemokines, CXCL11 was positively related to approximately all other chemokines except CCL14, CCL15, CCL16, CCL27, CCL28, CXCL6, and CXCL17 in almost all types of cancers. Moreover, elevated CXCL11 expression was related to increased immunosuppressive factors except for VTCN1 in almost all types of cancer except THYM (**Figure 10C**). Similar to immunosuppressive genes, CXCL11 expression was positively associated with most immunostimulatory genes in all cancers we analyzed except THYM (**Figure 11D**). Our data also showed that CXCL11 expression was positively related to all MHC genes in most cancers except DLBC (**Figure 11E**).

### GSEA of CXCL11 in KEGG and HALLMARK Pathways

**Figure 12:**
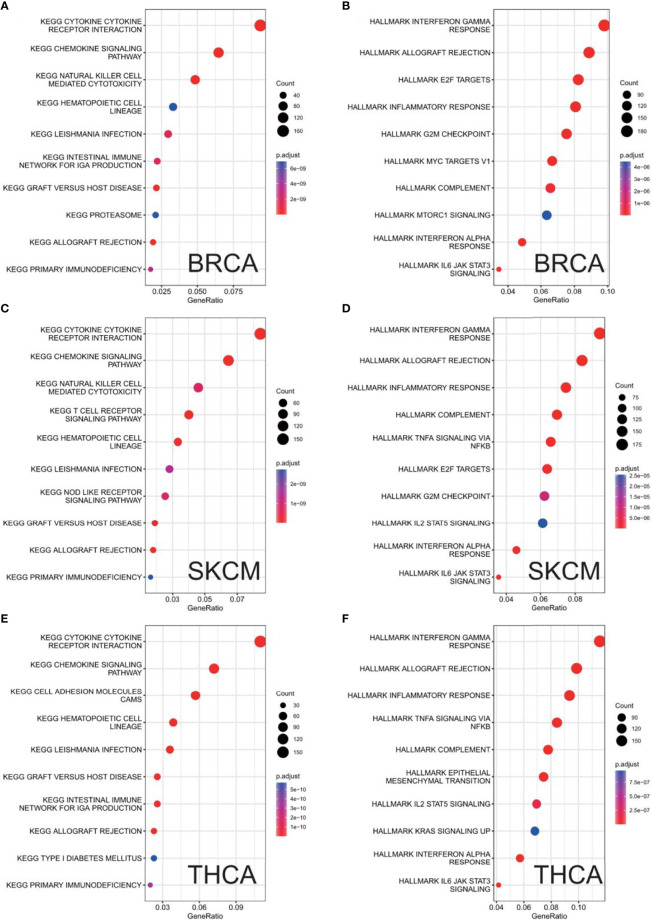
Results of GSEA. Different KEGG and HALLMARK pathways regulated in BRCA **(A, B)**, SKCM **(C, D)**, and THCA **(E, F)**.

Finally, GSEA was used to study the biological role of CXCX11 in different tumor tissues. Our data showed that the top 3 positively enriched KEGG pathways in the elevated expression of CXCL11 were cell adhesion molecules (CAMs), cytokine–cytokine receptor interactions, and leishmania infection. Interestingly, the three pathways were enriched in all 33 types of cancers. The HALLMARK enrichment term showed that CXCL11 expression was positively associated with processes including complement, IL6-JAK-STAT3 signaling, inflammatory response, interferon-α response, interferon-γ response, allograft rejection, TNF-a signaling *via* NF-κb, and IL2-STAT5 signaling in most of the cancers we analyzed. Here, the results of BRCA, SKCM, and THCA are shown in **Figure 12**.

## Discussion

This study first analyzed CXCL11 expression across all 33 cancers using emerging data from the TCGA and GTEx databases. The results showed that CXCL11 expression was significantly upregulated in ACC, BLCA, BRCA, CESC, CHOL, COAD, ESCA, GBM, HNSC, KIRC, LIHC, LUAD, OV, PCPG, PRAD, READ, SKCM, STAD, TGCT, THCA, UCEC, and UCS compared to nonnormal tissues (**Figure 1**).Our data was in agreement with previous study (30), that reported that CXCL11 expression was upregulated in COAD and READ. CXCL11 expression was differentially associated with tumor stage in certain types of cancers (**Figure 2**), while the association of upregulated CXCL11 expression with OS, DSS, DFI, or PFI was inconsistent in other types of cancers (**Figures 3**–**6**). CXCL11 overexpression was associated with poor prognosis in multiple types of cancers, such as LGG, while it was associated with good prognosis in multiple types of cancers, such as COAD, SKCM, KIRP, and OV. Time-dependent ROC curves for 1-, 3-, 5-, and 8-year OS showed the CXCL11 expression model and area under the curve (AUC) value (**Figure 7**). In this study, CXCL11 expression was associated with MMR genes, TMB, and MSI in certain cancers (**Figure 8**). Our results revealed that CXCL11 expression was positively related to stromal scores, immune scores, and ESTIMATE scores across cancers (**Figure 9**). Improving immunogenicity by altering the TME could be the future for cancer therapy (31), though the role of CXCL11 and its impact on the TME are not fully understood. Moreover, immune cells are an essential component of the TME. Therefore, we examined the correlation between CXCL11 expression and immune cell infiltration. The present study revealed the relationship between CXCL11 and tumor immune cells and found that CXCL11 significantly correlated with the infiltration levels of CD8+ T cells, T follicular helper cells, and MDSCs in most cancers (**Figure 10**). The correlations between CXCL11 expression and immunoregulation-related genes, chemokine receptors and other chemokines were evaluated. The results showed that there was a high correlation between CXCL11 expression and immunoregulation genes, chemokine receptors and other chemokines in almost all types of cancers. Moreover, most genes we analyzed showed a positive correlation with CXCL11 expression. (**Figure 11**). The KEGG and HALLMARK analyses suggested that CXCL11 was significantly associated with many immune-relevant signaling pathways (**Figure 12**).

The correlations of CXCL11 with various parameters (such as stage and survival) are different for different tumor types. By analyzing the relationship between CXCL11 expression and various parameters in different tumors, we found that changes in CXCL11 expression at different stages are also related to the relationship between CXCL11 and prognosis. CXCL11 expression is higher in the low stage in COAD (higher in stage I than stage IV, higher in stage II than stage III and IV) and SKCM (higher in stage I than stage II). The high expression of CXCL11 correspond to better prognosis outcomes in COAD (including longer DSS, DFI, PFI) and SKCM (including longer OS, DSS).CXCL11 expression is lower in the low stage in KIRP(including lower in stage I than stage III) and PAAD(lower in stage I than stage II). The low expression of CXCL11 corresponded to a better prognosis outcome in KIRP (longer DSS, DFI, and PFI) and PAAD (longer OS and DSS). CXCL11 expression is positively related to TMB but negatively related to MSI in BLCA, CESC, LUAD, and SKCM; the expression of CXCL11 is positively related to both TMB and MSI COAD; the expression of CXCL11 is negatively related to both TMB and MSI HNSC, TGCT.

In conclusion, our pan-cancer analyses of CXCL11 expression revealed that CXCL11 was differentially expressed in tumor and nontumoral tissues and at different tumor stages. This study also revealed the relationship between CXCL11 and clinical prognosis. Our results revealed that CXCL11 could act as an independent prognostic factor in many cancers. The different expression levels were associated with different clinical outcomes, which warrants further investigation of the specific role of CXCL11 in each cancer. CXCL11 was positively or negatively associated with TMB and MSI in different cancers. These results might provide a reference to shed light on the function of CXCL11 in tumorigenesis and development.

## Data Availability Statement

The original contributions presented in the study are included in the article/supplementary material. Further inquiries can be directed to the corresponding author.

## Author Contributions

All authors made substantial contributions to the conception and design, the acquisition of data, or the analysis and interpretation of data. All authors took part in drafting the article or revising it critically for important intellectual content. All authors agreed to the current journal submission. All authors approved the version to be published. All authors to be accountable for all aspects of the work.

## Funding

The article was supported by the Natural Science Foundation of China (81974377), the Scientific Research Project of the Education Department of Liaoning Province (JC2019017), 345 Talent Project of Shengjing Hospital (2019–2021), and the Outstanding Scientific Fund of Shengjing Hospital.

## Conflict of Interest

The authors declare that the research was conducted in the absence of any commercial or financial relationships that could be construed as a potential conflict of interest.

## Publisher’s Note

All claims expressed in this article are solely those of the authors and do not necessarily represent those of their affiliated organizations, or those of the publisher, the editors and the reviewers. Any product that may be evaluated in this article, or claim that may be made by its manufacturer, is not guaranteed or endorsed by the publisher.
